# Audiovestibular Dysfunction in Systemic Lupus Erythematosus Patients: A Systematic Review

**DOI:** 10.3390/diagnostics14151670

**Published:** 2024-08-01

**Authors:** Jiann-Jy Chen, Chih-Wei Hsu, Yen-Wen Chen, Tien-Yu Chen, Bing-Syuan Zeng, Ping-Tao Tseng

**Affiliations:** 1 Prospect Clinic for Otorhinolaryngology & Neurology, Kaohsiung 81166, Taiwan; jiannjy@yahoo.com.tw (J.-J.C.); kevinachen0527@gmail.com (Y.-W.C.); 2 Department of Otorhinolaryngology, E-Da Cancer Hospital, I-Shou University, Kaohsiung 80756, Taiwan; 3 Department of Psychiatry, Kaohsiung Chang Gung Memorial Hospital and Chang Gung University College of Medicine, Kaohsiung 83301, Taiwan; harwicacademia@gmail.com; 4 Department of Psychiatry, Tri-Service General Hospital, School of Medicine, National Defense Medical Center, Taipei 11490, Taiwan; verducciwol@gmail.com; 5 Institute of Brain Science, National Yang Ming Chiao Tung University, Taipei 11221, Taiwan; 6 Institute of Biomedical Sciences, National Sun Yat-sen University, Kaohsiung 80424, Taiwan; 7 Department of Internal Medicine, E-Da Cancer Hospital, I-Shou University, Kaohsiung 82445, Taiwan; 8 Department of Psychology, College of Medical and Health Science, Asia University, Taichung 413305, Taiwan; 9 Institute of Precision Medicine, National Sun Yat-sen University, Kaohsiung 804201, Taiwan

**Keywords:** systemic lupus erythematosus, cochleopathy, vestibular, sensorineural hearing loss, treatment

## Abstract

Audiovestibular dysfunction in patients with systemic lupus erythematosus has been underestimated for decades. Systemic lupus erythematosus can affect both the auditory and vestibular systems simultaneously. Several potential pathophysiological mechanisms behind systemic lupus erythematosus-related audiovestibular dysfunction have been proposed, including antibody-mediated immune responses, cell-mediated cytotoxicity, immune complex deposition in microvessels, central involvement in the audiovestibular pathway, and ototoxicity from medications used in systemic lupus erythematosus treatment. Currently available tests to evaluate audiovestibular function in systemic lupus erythematosus patients are neither specific nor sensitive. Nevertheless, there is no consensus regarding the efficacy of treatments for audiovestibular dysfunction in such patients. In this systematic review, we electronically searched the PubMed, Embase, ClinicalKey, Web of Science, and ScienceDirect platforms to find eligible articles. The first inspection date was on 29 December 2023 and the final update search date was on 11 June 2024. Further, we rated the quality of the included articles with Newcastle–Ottawa Scale. Based upon the aforementioned systematic review process, we have summarized the currently available evidence on the characteristics, pathophysiology, examination, and treatment of audiovestibular dysfunction related to systemic lupus erythematosus. Furthermore, we have proposed a specific steroid treatment protocol to manage audiovestibular dysfunction related to systemic lupus erythematosus. Audiovestibular dysfunction related to systemic lupus erythematosus may be responsive to adequate treatments, potentially allowing for reversibility if the disease is recognized and managed in a timely manner. Therefore, to provide clinically relevant evidence to clinicians, we have organized this literature review article to summarize the available evidence on the characteristics, pathophysiology, examination, and treatment of audiovestibular dysfunction in patients with systemic lupus erythematosus. Finally, based on our modified steroid treatment protocol, we would like to provide a new treatment strategy to clinicians to manage systemic lupus erythematosus-related audiovestibular dysfunction.

## 1. Introduction

Systemic lupus erythematosus (SLE) stands as one of the most prominent and severe autoimmune diseases, with an incidence rate ranging from 1.48 to 11.0 cases per 100,000 population worldwide [1,2], notably affecting females [3]. SLE is often accompanied by high comorbidity rates across various organs and systems [4], including hypertension (48%), depression (30%), hyperlipidemia (25%), osteoarthritis (25%), and osteoporosis (20%) [4].

The pathophysiology of SLE is multifaceted, involving genetic chromosomal alterations, hormonal and environmental factors, inflammatory stimulation, drug exposure, and interactions between the adaptive and innate immune systems [3]. Several autoantibodies have been implicated in the pathogenesis of SLE, including anti-dsDNA, anti-ssDNA, anti-nuclear antibodies, and antibodies to ribosomes or RNA polymerase, often accompanied by antiphospholipid antibodies [5]. These autoantibodies contribute not only to multiple solid organ involvement but also affect the hematologic and coagulation systems. For instance, circulating immune complexes in SLE patients may lead to vasculitis through excessive deposition in vessel tissues [3] or cause direct damage to inner ear tissues through autoantibody-mediated immune responses [6].

Recent reports have increasingly focused on the impact of autoimmune diseases on the audiovestibular system [7,8,9,10]. While some propose theoretically plausible etiologies [11,12], others lack acceptable pathophysiological explanations behind autoimmune diseases and audiovestibular system dysfunction [13]. However, only a few disease entities have histopathological evidence establishing a relationship between autoimmune reactions and the audiovestibular system, such as SLE [14]. Specifically, in patients with autoimmune-mediated hearing loss, serum autoantibodies may bind specifically to inner ear tissues [6], suggesting a humoral-type autoimmune process in the inner ear tissue [15]. Besides antibody-mediated pathophysiology, cell-mediated cytotoxicity toward inner ear tissues [15] and immune complex deposition in the microvessels of the audiovestibular organ [16] also play significant roles in the etiology of SLE-related audiovestibular dysfunction. This theoretical association between sensorineural hearing loss and SLE could be supported by one large-scale population-based study by Lin and colleagues. In their report, the authors demonstrated that the incidence of sensorineural hearing loss was 2.22-fold higher in patients with SLE than in the non-SLE subjects (6.52 vs. 2.93 per 10,000 person-years) [17].

Although there has been some argument regarding the necessity of audiovestibular involvement in SLE patients [18], researchers have recognized that sensorineural hearing loss or vestibular system dysfunction may serve as early signs (i.e., prodromal signs) in several connective tissue autoimmune diseases like SLE [19,20,21]. Previous reports have demonstrated impaired hearing thresholds across all frequencies except 2000 to 4000 Hz in SLE patients [22]. The course of hearing loss in SLE patients can be sudden-onset [23] or progressive [22]. As previous research has noted, the presence of SLE may be associated with the “premature aging” of the inner ear in young patients with SLE [24].

Distinct from other idiopathic hearing loss diseases, audiovestibular dysfunction related to SLE may respond to appropriate treatments [14,23,25], potentially allowing for reversibility if the disease is recognized and managed promptly. Therefore, to provide clinically relevant evidence to clinicians, the aim of this literature review article is to summarize the available evidence regarding the characteristics, pathophysiology, examination, and treatment for audiovestibular dysfunction in patients with SLE. To be specific, we summarized the typical presentation and recommended diagnostic tools of audiovestibular dysfunction in patients with SLE. Further, to overcome the currently unsatisfactory treatment option, we provided some favorable treatment recommendation choices and a modified steroid treatment protocol at the end of this systematic review.

## 2. Methods and Materials

**Figure 1:**
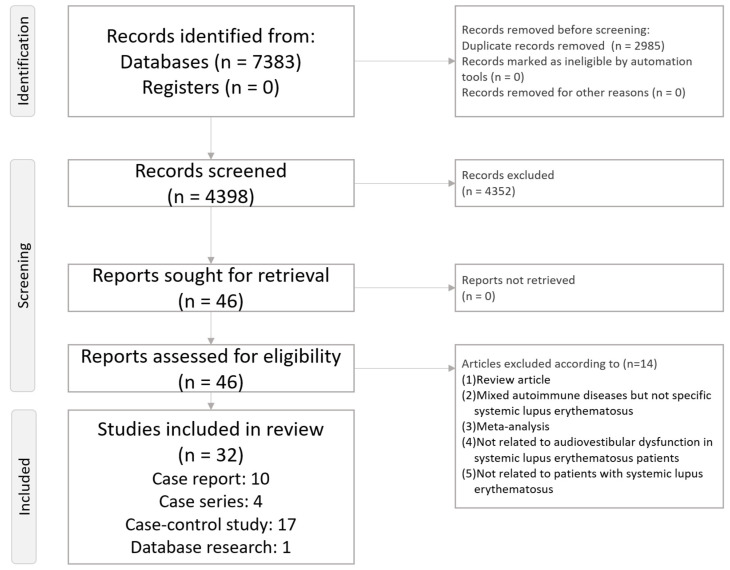
Flowchart of the whole systematic review procedure.

This systematic review follows the direction of Preferred Reporting Items for Systematic Review and Meta-Analysis (PRISMA) statement (Table S1 and Figure 1) [26]. The current systematic review had been registered on the INPLASY platform (INPLASY202460046, https://inplasy.com/inplasy-2024-6-0046/ accessed on 13 June 2024).

Figure 1 demonstrates a flowchart illustrating the procedure of the present systematic review.

### 2.1. Literature Search Strategy

This systematic review was conducted by electronically searching the PubMed, Embase, ClinicalKey, Web of Science, and ScienceDirect online platforms by Jiann-Jy Chen, Chih-Wei Hsu, and Yen-Wen Chen. The detailed search strategy and keywords used in each platforms are listed in Table S2. To be specific, we did not set any limitation to language during our search process. The first inspection date was on 29 December 2023 and the final update search date was on 11 June 2024. Additionally, a manual literature search was performed to scrutinize the reference lists of the included articles. The final update search date was 12 June 2024. If there was insufficient information available in the original paper, we would contact the corresponding authors via email to request data of interest. Jiann-Jy Chen, Chih-Wei Hsu, and Yen-Wen Chen conducted the full-text review process.

### 2.2. Inclusion and Exclusion Criteria

The current systematic review aims to focus on audiovestibular issues, including the characteristics, pathophysiology, examination, and treatment, in patients with SLE. Therefore, the inclusion criteria were as follows: (a) articles that examined the aforementioned audiovestibular issues in patients with SLE; (b) case reports/series, observational trials, case–control trials, or randomized controlled trials; and (c) articles recruiting patients with SLE.

The exclusion criteria were (a) articles not recruiting patients with SLE; (b) articles not related to information on the characteristics, pathophysiology, examination, or treatment related to audiovestibular dysfunction in patients with SLE; and (c) animal studies. Review articles were chosen to manually extract articles from their reference list. The excluded articles are listed in Table S3.

### 2.3. Article Screening Process

After all five databases were electronically searched with the aforementioned inclusion/exclusion criteria, all of the search items were screened by title and abstract. All of the eligible articles were downloaded and entered full-text examination. In this stage, duplicate articles would be removed manually and the other articles would be screened by full-text examination to determine whether they would enter final inclusion or not.

### 2.4. Data Extraction

Data extraction was performed by Ping-Tao Tseng, who conducted the full-text examination and extracted data on the characteristics, pathophysiology, examination, and treatment of patients with SLE.

### 2.5. Article Quality Grading

All the clinical studies were graded by Jiann-Jy Chen and Ping-Tao Tseng via the Newcastle–Ottawa Scale [27] (Table S4). To be specific, the Newcastle–Ottawa Scale consista of three domains, including Selection, Comparability, and Exposure, to rate the quality of a non-randomized trial. The overall quality of an indicated article was calculated by the total numbers of stars (*). The process of article quality rating was independently conducted by Jiann-Jy Chen and Ping-Tao Tseng. If there was any discrepancy between these two authors, the third author (Tien-Yu Chen) would be consulted to achieve consensus.

## 3. Results and Discussion

Overall, a total of 32 articles were included in the current systematic review. Among these 32 articles, 10 were case reports, 4 were case series, 17 were case–control studies, and 1 was database research (Table S5).

### 3.1. Vestibular System Involvement

#### 3.1.1. Characteristics

The prevalence of vestibular system involvement in SLE patients is estimated at around 70%, often presenting with complaints of vertigo and dizziness [22]. Vestibulopathy might be viewed as an early manifestation of SLE in clinical reports [28]. Reports vary widely regarding the prevalence of vertigo/balance disorders, ranging from 9% to 67% [20,29,30]. Despite this high prevalence, few studies have explored vestibular symptoms in SLE patients [20,31,32]. A significant proportion (31.0%) of SLE patients report comorbid aural symptoms, with subjects showing subtly higher creatinine levels and lower C3 levels [29]. Regarding comorbid migraine, not all reports support a relationship between migraine and vertigo/hearing loss in SLE patients [30]. Abnormal findings in electronystagmography are found in approximately 50% of SLE patients [20], with around 16.6% exhibiting vestibular hypofunction in caloric tests, indicating peripheral vertigo [30].

#### 3.1.2. Physiopathology

The potential linkage between SLE and vestibular system involvement may be associated with several factors. Firstly, complications of neuropsychiatric SLE syndromes, including cerebrovascular disease (2.2%), movement disorders (1.1%), sensorimotor polyneuropathy (29.22%), and cranial neuropathy (2.2%), may account for a significant portion of vestibular system dysfunction [33]. Secondly, dysfunctional cerebral blood flow has been observed in neuroimaging studies of SLE patients [34]. Thirdly, neuropathological studies support central nervous system involvement in SLE patients [35]. Fourthly, immune-mediated attacks on central nervous system elements have been identified in immunologic studies [36]. Finally, immunohistopathology in the temporal bone of SLE patients supports vestibular abnormalities [37], with a high prevalence of peripheral-type vestibular pathology [20]. In another temporal bone autopsy study, the authors noticed that there was significantly lower density of type I hair cells in the SLE patients than the healthy controls in the saccular macula, utricular macula, and three semicircular canals [38].

The potential linkage between vestibular dysfunction and SLE may involve humoral-type antibodies [15] and cell-mediated cytotoxicity [39] targeting inner ear antigens or immune complex deposition in the inner ear microcirculation [16]. In the report by Karatas and colleagues, the authors noticed a correlation between abnormal immune profiles and electronystagmography abnormalities in patients with systemic lupus erythematosus [20]. Additionally, positive results for antiphospholipid antibodies in SLE patients may increase the risk of Meniere’s disease, characterized by dizziness or vertigo [40]. In the report by Mouadeb and colleagues, the authors noticed that the prevalence rate of Meniere disease would achieve 64% in those systemic lupus erythematosus patients with positive antiphospholipid antibodies [40]. Microthrombus formation in labyrinthine vasculature is suggested as the pathogenesis of antiphospholipid antibodies [40]. However, among SLE patients with sensorineural hearing loss and antiphospholipid antibodies, additional clinical manifestations related to antiphospholipid antibodies, such as thrombocytopenia, are commonly reported [23]. Vestibular system damage could also be supported by the findings reported in a pathological study [37]. Pathological studies reveal vestibular system damage, with type I vestibular hair cell dysfunction being a common finding (29% of cases) [37], followed by vestibular fibrosis (6%) and hydrops (4%) [14].

#### 3.1.3. Examination

Systemic lupus erythematosus patients with vestibular system involvement may exhibit higher creatinine and lower C3 levels [29]. Abnormal findings in electronystagmography and caloric tests are also common in these patients [20,30]. However, these tests lack sensitivity and specificity for detecting vestibular system involvement in SLE. While histopathological examination in autopsy may aid in determining vestibular system involvement [14], it is clinically irrelevant due to the impracticality of performing such procedures on living patients.

### 3.2. Auditory System Involvement

#### 3.2.1. Characteristics

Auditory system involvement, characterized by unilateral or bilateral sensorineural hearing loss, is one of the most common otologic symptoms in SLE patients [14,41] and can sometimes be the initial manifestation of the disease [20]. The relative risk for sensorineural hearing loss is calculated as 3.7 in SLE patients compared to normal controls [19]. In recent meta-analyses, patients with SLE had significantly higher odds of hearing loss than the control group [42,43]. To be specific, there were significantly higher odds of sensorineural hearing loss but not conductive hearing loss in SLE patients than healthy controls [44]. Hearing thresholds in SLE patients are significantly higher than those in age-matched controls [22]. While most SLE patients with sensorineural hearing loss are asymptomatic (around 66%) [45], 15.6% may have definite sensorineural hearing loss [46], typically bilateral, symmetrical, and involving high frequencies [22,47,48]. Different from the typical features of asymmetry, fluctuation, and middle-frequency involvement in other autoimmune inner ear diseases [7], fluctuating patterns are not observed in SLE patients [22]. Additionally, the frequency of sensorineural hearing loss in SLE patients may be higher compared to other autoimmune inner ear diseases due to the often asymptomatic nature of hearing loss in SLE [45]. Some researchers suggest that hearing loss may be associated with a prolonged and exacerbated disease course, especially in younger patients [22]. While most studies report a similar percentage of SLE patients with unilateral or bilateral involvement [20,29], some suggest that bilateral involvement may occur in 100% of SLE patients with hearing impairment [19]. Impaired hearing thresholds may not always correlate with severity, disease duration, or autoantibody profile of SLE [19,22,49]. Progressive sensorineural hearing loss is not associated with anti-nuclear antibody or antiphospholipid antibody titers but may be linked to reduced C3 or C4 levels [30]. Approximately 83.3% of SLE patients with hearing loss exhibit reduced C3 or C4 levels, a phenomenon not observed in patients with Meniere’s disease [30]. However, around 27% of patients with sudden sensorineural hearing loss are associated with positive anti-cardiolipin antibody, suggesting an acute and sudden disease course [50].

No specific prodromal signs are present to detect early signs of sensorineural hearing loss in SLE patients, indicating a lack of alarming signs [19]. Hypoacusis, ear fullness, and tinnitus are the most prevalent subjective symptoms of impaired auditory system in SLE patients [29,46]. Despite impaired hearing, speech perception, distortion-product otoacoustic emissions, tympanogram, and acoustic reflexes may remain intact [21]. Conductive hearing loss is also found in SLE patients [21].

In addition to the impairment in the peripheral auditory pathway, Bruner and colleagues demonstrated an impairment in central auditory processing in SLE patients, especially those with neuropsychiatric disorders [51]. To be specific, SLE patients had significantly lower performance in temporal processing tests than healthy controls.

#### 3.2.2. Physiopathology

The main mechanisms of SLE-related inner ear damage include immune complex deposition and associated vascular dysfunction (e.g., vasculitis and microembolism), direct antibody/antigen reactions, cytotoxic action, retrocochlear or central involvement in the auditory pathway, and ototoxicity from medications used to treat SLE [14,22,52,53].

Vasculitis is one potential pathophysiology of audiology system involvement in SLE [22]. Free-radical-related cochlear pathology in the stria vascularis has been observed in SLE animal models [54]. Vasculitis may be mediated by immune complex deposition in the auditory artery, leading to reduced vessel caliber and decreased blood flow and oxygen supply [55]. Insufficient oxygen supply may stimulate the release of reactive oxygen species, causing impairment of hair cells and spiral ganglions, ultimately contributing to sensorineural hearing loss [56]. Stria vascularis atrophy (33%) and spiral ganglion degeneration (23%) are consistently reported in various studies [14]. Loss of outer hair cells and shrinkage of the stria vascularis may contribute to sensorineural hearing loss across all frequencies [57]. Dysfunctional arteries in the temporal bone may result from circulating immune complexes and consequent microinfarctions in the capillaries/arterioles [58]. Progressive vessel dysfunction may increase vessel resistance and lead to fibrotic changes in the stria vascularis [59]. Lack of response/prevention by steroid treatment may indicate a thrombotic rather than an inflammatory etiology of sensorineural hearing loss in SLE [23].

In addition to vascular-related otologic damage, cochlear hydrops and perisaccular deposition of immune complexes may contribute to hearing loss in SLE patients, especially those with positive anti-cardiolipin antibodies [60]. However, the prevalence of cochlear hydrops may be lower than vascular-related otologic damage [14].

Although the exact pathophysiology of auditory organ damage in autoimmune diseases like SLE is not fully understood, histopathological alterations have been observed in autopsy studies. Loss of hair cells, atrophy of the stria vascularis, and spiral ganglion damage have been reported in the temporal bone of SLE patients, supporting auditory tissue injury [61]. In severe cases, fibrotic changes in the round window membrane and inner ear tissue, replacement of whole sensorineural structures, and significant inflammatory infiltration in the cochlea have been observed [62]. Moderate to severe inner hair cell damage, especially of the outer hair cells, is frequently reported [14]. Inner ear involvement is most common in the middle and apical turns of the cochlea [14,37,61,62,63], while generalized involvement in all turns is relatively rare [61]. The pathophysiology may involve both direct autoantibody attack and cytotoxic damage by SLE [14]. Increased concentration and activity of autoantibodies in the perilymph may contribute to cytotoxic mechanisms and endolymph protein elevation, resulting in hair cell degeneration [64]. Additionally, an increase in auditory neural conduction has been observed in SLE patients, suggesting potential retrocochlear or central involvement in the auditory pathway [22].

Furthermore, medications used to treat SLE, such as chloroquine [65] and nonsteroidal anti-inflammatory drugs [52], may also play a role in sensorineural hearing impairment.

#### 3.2.3. Examination

While not specific or sensitive to SLE patients with auditory system involvement, several investigative tools can detect sensorineural hearing loss in these patients. Pure-tone audiometry can aid in early detection, especially of high-frequency predominant impairment [22]. In one recent report investigating the application of extended high-frequency audiometry in the early detection of sensorineural hearing loss in SLE patients, the authors demonstrated that about 70% of SLE patients have sensorineural hearing loss in extended high-frequency audiometry [66]. Further, there were statistically significant correlations between sensorineural hearing loss and age, disease activity and cryoglobulinemia [66]. Similar findings could be supported by another recent study, which revealed that the extended high-frequency audiometry was significantly impaired in SLE patients in comparison with healthy controls despite normal findings in the pure-tone audiometry [67].

Transiently evoked otoacoustic emissions (OAEs) have been found to detect cochlear outer hair cell dysfunction in SLE patients. To be specific, in the report by Cordeschi and colleagues, a statistically significant decrease in transiently evoked OAEs’ amplitude was found in comparison with healthy controls [68], which had an inverse correlation with the duration of disease [68]. Similar findings could be supported by the study by Karabulut and colleagues, in which the authors noticed both significantly different distortion-product OAEs and transiently evoked OAEs between SLE patients and healthy controls [69]. Speech perception, distortion-product otoacoustic emissions, tympanogram, and acoustic reflexes can help distinguish sensorineural hearing loss caused by SLE from other types of hearing loss [21]. Auditory brainstem response audiometry with a high stimulation rate may serve as a sensitive indicator for subclinical dysfunction in the central auditory system in autoimmune diseases [70], although its conclusive application has not been proven [71].

Decreased C3 or C4 levels may support hearing impairment in SLE patients [30,72]. Positive anti-cardiolipin antibody may help confirm diagnosis, especially in those with sudden-onset features [50]. Although there has been one report describing the association between low-density lipoprotein (LDL) levels and sensorineural hearing loss in SLE patients, the application of LDL in the diagnosis of sensorineural hearing loss in such patients remained unclear [73].

Finally, while histopathological examination in autopsy may help determine auditory system involvement [14], it is clinically irrelevant due to the difficulty of performing such procedures on living patients.

### 3.3. Treatment of Systemic Lupus Erythematosus-Related Vestibular and Auditory Dysfunction

Currently, there is no specific treatment available for vestibular or auditory impairment in SLE patients [14]. Instead, treatment primarily focuses on managing the underlying SLE, which typically involves modalities such as steroid therapy, plasmapheresis, anticoagulant therapy, cyclophosphamide, and monoclonal antibodies [14].

Steroid therapy, widely used in various autoimmune diseases and their complications, may be linked to SLE-related hearing loss through its anti-inflammatory, immunosuppressive, and anti-edema effects [14]. Steroid treatment could help to restore the capacity of otologic vessels via its anti-inflammatory and anti-edema effects [14]. Further, the side effect of the increased systemic blood pressure related to steroid treatment could also help in increasing the blood perfusion in the auditory artery [14]. However, while steroids are commonly used in SLE patients with hearing impairment, their efficacy in treating hearing loss specifically related to SLE remains uncertain [19,47,74]. Few reports have demonstrated unsatisfactory efficacy of steroids in remedying hearing impairment in these patients [25,58,75]. However, high-dosage intravenous steroid pulse therapy might have a therapeutic effect on the sensorineural hearing function in SLE patients, especially the sudden-onset form [76]. On the other hand, one case report demonstrated the beneficial effect of continuous high-dosage steroid treatment (i.e., prednisone 60 mg/day) with additional oral azathioprine (200 mg/day) on the pure-tone thresholds, the speech reception threshold, and word recognition scores in a patient with SLE [77]. Similarly, the beneficial effect of continuous high-dosage steroid treatment (i.e., 40 mg/day prednisone for 14 days) in progressive sensorineural hearing loss in SLE patients has been reported in another report [78].

Plasmapheresis, involving extracorporeal exchange of blood plasma or components, has been used to manage various SLE-related complications. Case reports suggest that plasmapheresis may be effective in restoring hearing loss refractory to steroid treatment [25,75]. Plasmapheresis may improve oxygen concentration in the inner ear and provide immunomodulatory effects [14].

Anticoagulants (i.e., low-molecular-weight heparin or warfarin) plus continuous oral steroids at 60 mg/day have been considered as an alternative treatment option for those patients with systemic lupus erythematosus and positive anti-cardiolipin antibody. In the report by Green, L. and Miller, E.B. (2001), the authors presented a case who had poor response to oral steroid monotherapy but improved after additional anticoagulant therapy [23]. However, in their report, that case’s hearing loss did not recover despite improvements in laboratory data. This therapy may improve blood fluidity and revascularization in the auditory circulation system, potentially benefiting patients with SLE-related hearing loss [14].

Cyclophosphamide, recommended as an adjunct to steroid treatment to reduce autoimmune activity, has been associated with improvement in the pure-tone average threshold in some SLE patients [79]. However, its efficacy may vary among individuals [58,80].

Other regimens, such as monoclonal antibody treatment (i.e., rituximab or epratuzumab), have been applied as a new option to manage SLE and its complications [81]. Ten-day hydrochlorothiazide (50 mg/day) has been found to restore hearing function in sudden sensorineural hearing loss in a patient with lupus erythematosus plus antiphospholipid syndrome [82].

#### Temporary Protocol of Steroid Treatment to Manage SLE-Related Audiovestibular Dysfunction

**Figure 2:**
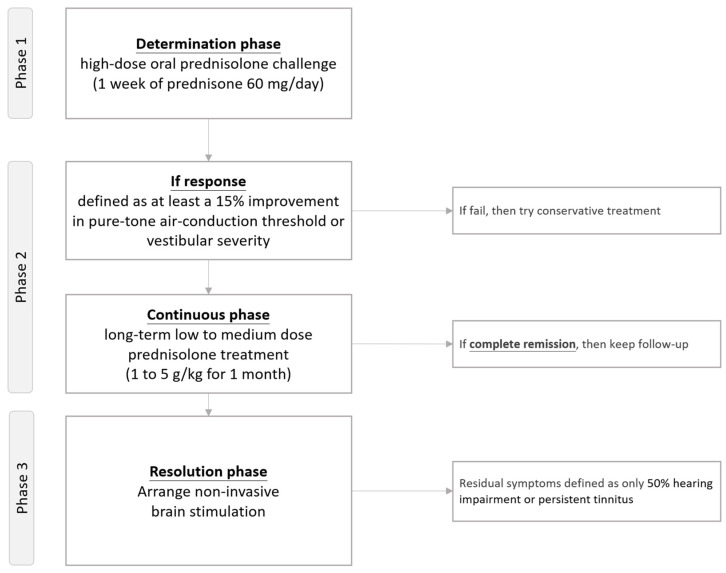
Flowchart of the modified steroid treatment protocol to manage systemic lupus erythematosus-related audiovestibular dysfunction.

From our unpublished preliminary data [83], we have developed a modified steroid treatment protocol (Figure 2) specifically for managing SLE-related audiovestibular dysfunction, distinct from other autoimmune inner ear diseases. The indication of this protocol was to manage SLE patients with comorbid audiovestibular dysfunction. The primary result was the restoration of hearing function or improvement of vestibular symptoms. This protocol, based on Alexander’s protocol for various autoimmune inner ear diseases, consists of a three-phase trial [84].

Figure 2 demonstrates the modified steroid treatment protocol, which focuses on the management of audiovestibular dysfunction related to systemic lupus erythematosus.

In our protocol, we arranged a three-phase trial. In phase 1, patients undergo a high-dose oral prednisolone trial (1 week of prednisone 60 mg/day) to determine their response to steroid treatment. A response is defined as at least a 15% improvement in pure-tone air-conduction threshold or vestibular severity. Following phase 1, patients begin long-term low- to medium-dose prednisolone treatment (1 to 5 g/kg for 1 month) in phase 2. Finally, in phase 3, patients with residual symptoms (50% hearing impairment or persistent tinnitus) undergo non-invasive brain stimulation [85,86].

## 4. Conclusions

This review article has synthesized the current knowledge about hearing impairment related to SLE. Through the available evidence, one undeniable fact emerges: unrecognized SLE-related hearing impairment can ultimately lead to hearing loss and diminish quality of life. As mentioned earlier, the absence of specific prodromal signs underscores the importance of clinician being aware of the potential risk of vestibular/auditory system involvement in asymptomatic SLE patients. Although most SLE patients with sensorineural hearing loss may not exhibit symptoms, a significant proportion may ultimately receive a diagnosis of hearing loss. Certain audiometric tests can aid in the early detection of hearing impairment in SLE patients. Therefore, utilizing these tools, routine check-ups for hearing ability should be conducted for SLE patients to potentially halt and prevent further hearing deterioration.

Moreover, unlike other idiopathic hearing loss diseases, audiovestibular dysfunction associated with SLE may respond to appropriate treatments, potentially being reversible if promptly recognized and managed. Therefore, we strongly advocate for early and routine screening of audiovestibular function in SLE patients, even during asymptomatic stages.

## Data Availability

The current systematic review had been registered in INPLASY platform (INPLASY202460046, https://inplasy.com/inplasy-2024-6-0046/ accessed on 13 June 2024).

## Supplementary Materials

The following supporting information can be downloaded at: https://www.mdpi.com/article/10.3390/diagnostics14151670/s1, Table S1: PRISMA 2020 checklist of current systematic review; Table S2: Keyword and search results in each database; Table S3: Excluded studies and reason; Table S4: Newcastle-Ottawa Scale and Characteristics for the Included Trial; Table S5: Summary of the included study [10,14,17,18,19,20,21,22,23,26,28,29,30,31,37,38,41,42,43,44,45,46,47,48,49,51,53,55,61,63,66,67,68,69,72,73,76,77,78,80,82,87,88,89,90,91,92].

## References

[1] Magro R., Borg A.A. (2018). Characterisation of Patients with Systemic Lupus Erythematosus in Malta: A Population Based Cohort Cross-Sectional Study. Biomed. Res. Int..

[2] Anstey N.M., Bastian I., Dunckley H., Currie B.J. (1993). Systemic lupus erythematosus in Australian aborigines: High prevalence, morbidity and mortality. Aust. N. Z. J. Med..

[3] Lehman T., Nuruzzaman F., Taber S., Cimaz R., Lehman T. (2016). Chapter 8—Systemic Lupus Erythematosus: Etiology, Pathogenesis, Clinical Manifestations, and Management. Handbook of Systemic Autoimmune Diseases.

[4] Albrecht K., Redeker I., Aringer M., Marschall U., Strangfeld A., Callhoff J. (2021). Comorbidity and healthcare utilisation in persons with incident systemic lupus erythematosus followed for 3 years after diagnosis: Analysis of a claims data cohort. Lupus Sci. Med..

[5] Suzuki N., Mihara S., Sakane T. (1997). Development of pathogenic anti-DNA antibodies in patients with systemic lupus erythematosus. FASEB J. Off. Publ. Fed. Am. Soc. Exp. Biol..

[6] Arnold W., Pfaltz R., Altermatt H.J. (1985). Evidence of serum antibodies against inner ear tissues in the blood of patients with certain sensorineural hearing disorders. Acta Otolaryngol..

[7] Ruckenstein M.J. (2004). Autoimmune inner ear disease. Curr. Opin. Otolaryngol. Head. Neck Surg..

[8] Mathews J., Rao S., Kumar B.N. (2003). Autoimmune sensorineural hearing loss: Is it still a clinical diagnosis?. J. Laryngol. Otol..

[9] Ralli M., Di Stadio A., De Virgilio A., Croce A., de Vincentiis M. (2018). Autoimmunity and Otolaryngology Diseases. J. Immunol. Res..

[10] Stone J.H., Francis H.W. (2000). Immune-mediated inner ear disease. Curr. Opin. Rheumatol..

[11] Colletti V., Fiorino F.G., Bruni L., Biasi D. (1997). Middle ear mechanics in subjects with rheumatoid arthritis. Audiology.

[12] Frade C., Martin C. (1998). Diagnostic value of the multifrequency tympanometry in active rheumatoid arthritis. Auris Nasus Larynx.

[13] Sun X.M., Zhuang S.M., Xiao Z.W., Luo J.Q., Long Z., Lan L.C., Zhang H.Q., Zhang G.P. (2022). Autoimmune thyroiditis in patients with sudden sensorineural hearing loss. Laryngoscope Investig. Otolaryngol..

[14] Di Stadio A., Ralli M. (2017). Systemic Lupus Erythematosus and hearing disorders: Literature review and meta-analysis of clinical and temporal bone findings. J. Int. Med. Res..

[15] Harris J.P., Sharp P.A. (1990). Inner ear autoantibodies in patients with rapidly progressive sensorineural hearing loss. Laryngoscope.

[16] Veldman J.E., Roord J.J., O’Connor A.F., Shea J.J. (1984). Autoimmunity and inner ear disorders: An immune-complex mediated sensorineural hearing loss. Laryngoscope.

[17] Lin C., Lin S.W., Weng S.F., Lin Y.S. (2013). Risk of sudden sensorineural hearing loss in patients with systemic lupus erythematosus: A population-based cohort study. Audiol. Neurootol..

[18] Garcia-Berrocal J.R., De Diego B., Roldan-Fidalgo A., Yebra-Bango M., Millan I., Trinidad A., Ramirez-Camacho R. (2013). Young systemic lupus erythematosus patients with no hearing involvement: 10-year follow up. J. Laryngol. Otol..

[19] Abbasi M., Yazdi Z., Kazemifar A.M., Bakhsh Z.Z. (2013). Hearing loss in patients with systemic lupus erythematosus. Glob. J. Health Sci..

[20] Karatas E., Onat A.M., Durucu C., Baglam T., Kanlikama M., Altunoren O., Buyukhatipoglu H. (2007). Audiovestibular disturbance in patients with systemic lupus erythematosus. Otolaryngol. Head. Neck Surg..

[21] Rahne T., Clauss F., Plontke S.K., Keysser G. (2017). Prevalence of hearing impairment in patients with rheumatoid arthritis, granulomatosis with polyangiitis (GPA, Wegener’s granulomatosis), or systemic lupus erythematosus. Clin. Rheumatol..

[22] Maciaszczyk K., Durko T., Waszczykowska E., Erkiert-Polguj A., Pajor A. (2011). Auditory function in patients with systemic lupus erythematosus. Auris Nasus Larynx.

[23] Green L., Miller E.B. (2001). Sudden sensorineural hearing loss as a first manifestation of systemic lupus erythematosus: Association with anticardiolipin antibodies. Clin. Rheumatol..

[24] Andonopoulos A.P., Naxakis S., Goumas P., Lygatsikas C. (1995). Sensorineural hearing disorders in systemic lupus erythematosus. A controlled study. Clin. Exp. Rheumatol..

[25] Hamblin T.J., Mufti G.J., Bracewell A. (1982). Severe deafness in systemic lupus erythematosus: Its immediate relief by plasma exchange. Br. Med. J. Clin. Res. Ed..

[26] Page M.J., McKenzie J.E., Bossuyt P.M., Boutron I., Hoffmann T.C., Mulrow C.D., Shamseer L., Tetzlaff J.M., Akl E.A., Brennan S.E. (2021). The PRISMA 2020 statement: An updated guideline for reporting systematic reviews. Bmj.

[27] Wells G.A., Shea B., O’Connell D., Peterson J., Welch V., Losos M., Tugwell P. The Newcastle-Ottawa Scale (NOS) for Assessing the Quality of Nonrandomised Studies in Meta-Analyses. https://www.ohri.ca/programs/clinical_epidemiology/oxford.asp.

[28] Kim J.G., Lee S.U., Lee C.N., Yu S.W., Park K.W., Kim J.S. (2020). Bilateral vestibulopathy as an early manifestation of systemic lupus erythematosus. J. Neurol..

[29] Sperling N.M., Tehrani K., Liebling A., Ginzler E. (1998). Aural symptoms and hearing loss in patients with lupus. Otolaryngol. Head. Neck Surg..

[30] Batuecas-Caletrio A., del Pino-Montes J., Cordero-Civantos C., Calle-Cabanillas M.I., Lopez-Escamez J.A. (2013). Hearing and vestibular disorders in patients with systemic lupus erythematosus. Lupus.

[31] Liao C.H., Yang Y.H., Chiang B.L. (2003). Systemic lupus erythematosus with presentation as vertigo and vertical nystagmus: Report of one case. Acta Paediatr. Taiwan..

[32] Hughes G.B., Moscicki R., Barna B.P., San Martin J.E. (1994). Laboratory diagnosis of immune inner ear disease. Am. J. Otol..

[33] Brey R.L., Holliday S.L., Saklad A.R., Navarrete M.G., Hermosillo-Romo D., Stallworth C.L., Valdez C.R., Escalante A., del Rincon I., Gronseth G. (2002). Neuropsychiatric syndromes in lupus: Prevalence using standardized definitions. Neurology.

[34] Sibbitt W.L., Sibbitt R.R., Brooks W.M. (1999). Neuroimaging in neuropsychiatric systemic lupus erythematosus. Arthritis Rheum..

[35] Ellis S.G., Verity M.A. (1979). Central nervous system involvement in systemic lupus erythematosus: A review of neuropathologic findings in 57 cases, 1955–1977. Semin. Arthritis Rheum..

[36] Kurki P., Helve T., Dahl D., Virtanen I. (1986). Neurofilament antibodies in systemic lupus erythematosus. J. Rheumatol..

[37] Fukushima N., Fukushima H., Cureoglu S., Schachern P.A., Paparella M.M. (2006). Hearing loss associated with systemic lupus erythematosus: Temporal bone histopathology. Otol. Neurotol..

[38] Kariya S., Hizli O., Kaya S., Hizli P., Nishizaki K., Paparella M.M., Cureoglu S. (2015). Histopathologic Findings in Peripheral Vestibular System from Patients with Systemic Lupus Erythematosus: A Human Temporal Bone Study. Otol. Neurotol..

[39] Bernstein J.M., Bernstein J., Ogra P. (1987). The immunobiology of autoimmune disease of the inner ear. Immunology of the Ear.

[40] Mouadeb D.A., Ruckenstein M.J. (2005). Antiphospholipid inner ear syndrome. Laryngoscope.

[41] Polanski J.F., Tanaka E.A., Barros H., Chuchene A.G., Miguel P.T.G., Skare T.L. (2021). Chloroquine, Hydroxychloroquine and Hearing Loss: A Study in Systemic Lupus Erythematosus Patients. Laryngoscope.

[42] Yuen E., Fried J., Nguyen S.A., Rizk H.G., Ward C., Meyer T.A. (2021). Hearing loss in patients with systemic lupus erythematosus: A systematic review and meta-analysis. Lupus.

[43] Li X., Cao Z., Chen F., Yang D., Zhao F. (2023). Sensorineural Hearing Loss in Autoimmune Diseases: A Systematic Review and Meta-analysis. J. Int. Adv. Otol..

[44] Paraschou V., Chaitidis N., Papadopoulou Z., Theocharis P., Siolos P., Festas C. (2021). Association of systemic lupus erythematosus with hearing loss: A systemic review and meta-analysis. Rheumatol. Int..

[45] Roverano S., Cassano G., Paira S., Chiavarini J., Graf C., Rico L., Heredia C. (2006). Asymptomatic sensorineural hearing loss in patients with systemic lupus erythematosus. J. Clin. Rheumatol..

[46] Gomides A.P., do Rosario E.J., Borges H.M., Gomides H.H., de Padua P.M., Sampaio-Barros P.D. (2007). Sensorineural dysacusis in patients with systemic lupus erythematosus. Lupus.

[47] Jimenez-Alonso J., Gutierrez-Cabello F., Castillo J.L., Sabio J.M., Hidalgo-Tenorio C., Leon L., Grupo Lupus Virgen de las Nieves (2002). Ear involvement in systemic lupus erythematosus patients: A comparative study. J. Laryngol. Otol..

[48] Riera J.L., Del R.M.M., Musuruana J.L., Cavallasca J.A. (2020). Sudden Sensorineural Hearing Loss in Systemic Lupus Erythematosus and Antiphospholipid Syndrome: A Clinical Review. Curr. Rheumatol. Rev..

[49] Kastanioudakis I., Ziavra N., Voulgari P.V., Exarchakos G., Skevas A., Drosos A.A. (2002). Ear involvement in systemic lupus erythematosus patients: A comparative study. J. Laryngol. Otol..

[50] Toubi E., Ben-David J., Kessel A., Podoshin L., Golan T.D. (1997). Autoimmune aberration in sudden sensorineural hearing loss: Association with anti-cardiolipin antibodies. Lupus.

[51] Bruner A.P., Sato E.I., Pereira L.D. (2009). Central auditory processing in patients with systemic lupus erythematosus. Acta Reumatol. Port..

[52] Jung T.T., Rhee C.K., Lee C.S., Park Y.S., Choi D.C. (1993). Ototoxicity of salicylate, nonsteroidal antiinflammatory drugs, and quinine. Otolaryngol. Clin. N. Am..

[53] Fernandes M.R.N., Soares D.B.R., Thien C.I., Carneiro S. (2018). Hydroxychloroquine ototoxicity in a patient with systemic lupus erythematosus. An. Bras. Dermatol..

[54] Ruckenstein M.J., Keithley E.M., Bennett T., Powell H.C., Baird S., Harris J.P. (1999). Ultrastructural pathology in the stria vascularis of the MRL-Fasl(lpr) mouse. Hear. Res..

[55] Rahne T., Plontke S., Keysser G. (2020). Vasculitis and the ear: A literature review. Curr. Opin. Rheumatol..

[56] Ralli M., D’Aguanno V., Di Stadio A., De Virgilio A., Croce A., Longo L., Greco A., de Vincentiis M. (2018). Audiovestibular Symptoms in Systemic Autoimmune Diseases. J. Immunol. Res..

[57] Triplett J.D., Buzzard K.A., Lubomski M., Riminton D.S., Barnett M.H., Welgampola M.S., Halmagyi G.M., Nguyen M., Landau K., Lee A.G. (2019). Immune-mediated conditions affecting the brain, eye and ear (BEE syndromes). J. Neurol. Neurosurg. Psychiatry.

[58] Caldarelli D.D., Rejowski J.E., Corey J.P. (1986). Sensorineural hearing loss in lupus erythematosus. Am. J. Otol..

[59] Johnsson L.G. (1973). Vascular pathology in the human inner ear. Adv. Otorhinolaryngol..

[60] Bouman H., Klis S.F., de Groot J.C., Huizing E.H., Smoorenburg G.F., Veldman J.E. (1998). Induction of endolymphatic hydrops in the guinea pig by perisaccular deposition of sepharose beads carrying and not carrying immune complexes. Hear. Res..

[61] Sone M., Schachern P.A., Paparella M.M., Morizono N. (1999). Study of systemic lupus erythematosus in temporal bones. Ann. Otol. Rhinol. Laryngol..

[62] Yoon T.H., Paparella M.M., Schachern P.A. (1989). Systemic vasculitis: A temporal bone histopathologic study. Laryngoscope.

[63] Kariya S., Kaya S., Hizli O., Hizli P., Nishizaki K., Paparella M.M., Cureoglu S. (2016). Cochlear Histopathologic Findings in Patients With Systemic Lupus Erythematosus: A Human Temporal Bone Study. Otol. Neurotol..

[64] Barna B.P., Hughes G.B. (1988). Autoimmunity and otologic disease: Clinical and experimental aspects. Clin. Lab. Med..

[65] Bernard P. (1985). Alterations of auditory evoked potentials during the course of chloroquine treatment. Acta Otolaryngol..

[66] Lasso de la Vega M., Villarreal I.M., Lopez Moya J., Garcia-Berrocal J.R. (2017). Extended high frequency audiometry can diagnose sub-clinic involvement in a seemingly normal hearing systemic lupus erythematosus population. Acta Otolaryngol..

[67] Chen H., Wang F., Yang Y., Hua B., Wang H., Chen J., Feng X. (2022). Characteristics of Hearing Loss in Patients with Systemic Lupus Erythematosus. J. Clin. Med..

[68] Cordeschi S., Salvinelli F., D’Ascanio L. (2004). Sensorineural hearing impairment in systemic lupus erythematosus: Sudden or progressive?. Clin. Exp. Rheumatol..

[69] Karabulut H., Dagli M., Ates A., Karaaslan Y. (2010). Results for audiology and distortion product and transient evoked otoacoustic emissions in patients with systemic lupus erythematosus. J. Laryngol. Otol..

[70] Fradis M., Podoshin L., Ben-David J., Statter P., Pratt H., Nahir M. (1989). Brainstem auditory evoked potentials with increased stimulus rate in patients suffering from systemic lupus erythematosus. Laryngoscope.

[71] Borton T.E., Eby T.L., Ball E.V., Nolen B.L., Bradley E.L. (1992). Stimulus repetition rate effect on the auditory brainstem response in systemic lupus erythematosus. Laryngoscope.

[72] Tan C.L., Yahaya M.H., Ahmad N.S., Lim C.H. (2020). Macrophage activation syndrome as an initial presentation of systemic lupus erythematosus with sensorineural hearing loss in a young male patient. BMJ Case Rep..

[73] Ferrari A.L., Calonga L., Lapa A.T., Postal M., Sinicato N.A., Pelicari K.O., Peres F.A., Valente J.P., Soki M., Appenzeller S. (2016). Low-Density Lipoprotein Cholesterol Is Associated With Asymptomatic Sensorineural Hearing Loss in Patients With Systemic Lupus Erythematosus. J. Clin. Rheumatol..

[74] Bowman C.A., Linthicum F.H., Nelson R.A., Mikami K., Quismorio F. (1986). Sensorineural hearing loss associated with systemic lupus erythematosus. Otolaryngol. Head. Neck Surg..

[75] Kobayashi S., Fujishiro N., Sugiyama K. (1992). Systemic lupus erythematosus with sensorineural hearing loss and improvement after plasmapheresis using the double filtration method. Intern. Med..

[76] Chawki S., Aouizerate J., Trad S., Prinseau J., Hanslik T. (2016). Bilateral sudden sensorineural hearing loss as a presenting feature of systemic lupus erythematosus: Case report and brief review of other published cases. Medicine.

[77] Khalidi N.A., Rebello R., Robertson D.D. (2008). Sensorineural hearing loss in systemic lupus erythematosus: Case report and literature review. J. Laryngol. Otol..

[78] Bullington M., Davies G., MacDonald C.B. (2019). Reversible Sensorineural Hearing Loss Resulting from Hypertrophic Pachymeningitis in Systemic Lupus Erythematosus: A Case Report. OTO Open.

[79] Kataoka H., Takeda T., Nakatani H., Saito H. (1995). Sensorineural hearing loss of suspected autoimmune etiology: A report of three cases. Auris Nasus Larynx.

[80] Girasoli L., Cazzador D., Padoan R., Nardello E., Felicetti M., Zanoletti E., Schiavon F., Bovo R. (2018). Update on Vertigo in Autoimmune Disorders, from Diagnosis to Treatment. J. Immunol. Res..

[81] Dasgupta S., Dasgupta S. (2017). Therapeutic Interventions of Tissue Specific Autoimmune Onset in Systemic Lupus Erythematosus. Mini Rev. Med. Chem..

[82] Compadretti G.C., Brandolini C., Tasca I. (2005). Sudden sensorineural hearing loss in lupus erythematosus associated with antiphospholipid syndrome: Case report and review. Ann. Otol. Rhinol. Laryngol..

[83] Chen J.J., Tseng P.T. (2024). The efficacy of a modified steroid treatment protocol in SLE-related audiovestibular dysfunction. Prospect Clinic for Otorhinolaryngology & Neurology, Kaohsiung City, Taiwan.

[84] Alexander T.H., Weisman M.H., Derebery J.M., Espeland M.A., Gantz B.J., Gulya A.J., Hammerschlag P.E., Hannley M., Hughes G.B., Moscicki R. (2009). Safety of high-dose corticosteroids for the treatment of autoimmune inner ear disease. Otol. Neurotol..

[85] Chen J.J., Zeng B.S., Wu C.N., Stubbs B., Carvalho A.F., Brunoni A.R., Su K.P., Tu Y.K., Wu Y.C., Chen T.Y. (2020). Association of Central Noninvasive Brain Stimulation Interventions With Efficacy and Safety in Tinnitus Management: A Meta-analysis. JAMA Otolaryngol. Head. Neck Surg..

[86] Chen J.J., Zeng B.Y., Lui C.C., Chen T.Y., Chen Y.W., Tseng P.T. (2022). Pfizer-BioNTech COVID-19 vaccine associated tinnitus and treatment with transcranial magnetic stimulation. QJM.

[87] Frejo L., Lopez-Escamez J.A. (2022). Cytokines and Inflammation in Meniere Disease. Clin. Exp. Otorhinolaryngol..

[88] Poshattiwar R.S., Acharya S., Shukla S., Kumar S. (2023). Neurological Manifestations of Connective Tissue Disorders. Cureus.

[89] Crincoli V., Piancino M.G., Iannone F., Errede M., Di Comite M. (2020). Temporomandibular Disorders and Oral Features in Systemic Lupus Erythematosus Patients: An Observational Study of Symptoms and Signs. Int. J. Med. Sci..

[90] Xie S., Ning H., She Y., Jing Q., Jiang Q., Zhang Y., Mei L., Feng Y., Wu X. (2020). Effect of systemic lupus erythematosus and rheumatoid arthritis on sudden sensorineural hearing loss. Laryngoscope.

[91] Motoyama R., Higuchi T., Hirahara S., Konda N., Yamada R., Watanabe K., Fujisaki M., Yamaguchi R., Katsumata Y., Kawaguchi Y. (2023). A case of systemic lupus erythematosus having concurrent Evans syndrome and acquired thrombotic thrombocytopenic purpura. Mod. Rheumatol. Case Rep..

[92] Gazquez I., Soto-Varela A., Aran I., Santos S., Batuecas A., Trinidad G., Perez-Garrigues H., Gonzalez-Oller C., Acosta L., Lopez-Escamez J.A. (2011). High prevalence of systemic autoimmune diseases in patients with Meniere’s disease. PLoS ONE.

