# Supplementary material for: Audiovestibular Dysfunction in Systemic Lupus Erythematosus Patients: A Systematic Review
Source: Diagnostics (Basel). 2024 Aug 1;14(15):1670. doi: 10.3390/diagnostics14151670 (PMC11311441; doi:10.3390/diagnostics14151670)
Supplement: Supplementary file 1 [file diagnostics-14-01670-s001.zip › diagnostics-3069148-supplementary.pdf]

Table S1: PRISMA 2020 checklist of current systematic review

Section and Topic	Item #	Checklist item	Page where item is reported
**TITLE**			
Title	1	Identify the report as a systematic review.	1
**ABSTRACT**			
Abstract	2	See the PRISMA 2020 for Abstracts checklist.	3
**INTRODUCTION**			
Rationale	3	Describe the rationale for the review in the context of existing knowledge.	4-5
Objectives	4	Provide an explicit statement of the objective(s) or question(s) the review addresses.	4-5
**METHODS**			
Eligibility criteria	5	Specify the inclusion and exclusion criteria for the review and how studies were grouped for the syntheses.	6-7
Information sources	6	Specify all databases, registers, websites, organisations, reference lists and other sources searched or consulted to identify studies. Specify the date when each source was last searched or consulted.	6-7
Search strategy	7	Present the full search strategies for all databases, registers and websites, including any filters and limits used.	6-7
Selection process	8	Specify the methods used to decide whether a study met the inclusion criteria of the review, including how many reviewers screened each record and each report retrieved, whether they worked independently, and if applicable, details of automation tools used in the process.	6-7
Data collection process	9	Specify the methods used to collect data from reports, including how many reviewers collected data from each report, whether they worked independently, any processes for obtaining or confirming data from study investigators, and if applicable, details of automation tools used in the process.	6-7
Data items	10a	List and define all outcomes for which data were sought. Specify whether all results that were compatible with each outcome domain in each study were sought (e.g. for all measures, time points, analyses), and if not, the methods used to decide which results to collect.	6-7
	10b	List and define all other variables for which data were sought (e.g. participant and intervention characteristics, funding sources). Describe any assumptions made about any missing or unclear information.	6-7
Study risk of bias assessment	11	Specify the methods used to assess risk of bias in the included studies, including details of the tool(s) used, how many reviewers assessed each study and whether they worked independently, and if applicable, details of automation tools used in the process.	6-7
Effect measures	12	Specify for each outcome the effect measure(s) (e.g. risk ratio, mean difference) used in the synthesis or presentation of results.	6-7
Synthesis methods	13a	Describe the processes used to decide which studies were eligible for each synthesis (e.g. tabulating the study intervention characteristics and comparing against the planned groups for each synthesis (item #5)).	Not done
	13b	Describe any methods required to prepare the data for presentation or synthesis, such as handling of missing summary statistics, or data conversions.	Not done

Section and Topic	Item #	Checklist item	Page where item is reported
	13c	Describe any methods used to tabulate or visually display results of individual studies and syntheses.	Not done
	13d	Describe any methods used to synthesize results and provide a rationale for the choice(s). If meta-analysis was performed, describe the model(s), method(s) to identify the presence and extent of statistical heterogeneity, and software package(s) used.	Not done
	13e	Describe any methods used to explore possible causes of heterogeneity among study results (e.g. subgroup analysis, meta-regression).	Not done
	13f	Describe any sensitivity analyses conducted to assess robustness of the synthesized results.	Not done
Reporting bias assessment	14	Describe any methods used to assess risk of bias due to missing results in a synthesis (arising from reporting biases).	6-7
Certainty assessment	15	Describe any methods used to assess certainty (or confidence) in the body of evidence for an outcome.	6-7
**RESULTS**			
Study selection	16a	Describe the results of the search and selection process, from the number of records identified in the search to the number of studies included in the review, ideally using a flow diagram.	8-9
	16b	Cite studies that might appear to meet the inclusion criteria, but which were excluded, and explain why they were excluded.	8-9
Study characteristics	17	Cite each included study and present its characteristics.	8-9
Risk of bias in studies	18	Present assessments of risk of bias for each included study.	8-9
Results of individual studies	19	For all outcomes, present, for each study: (a) summary statistics for each group (where appropriate) and (b) an effect estimate and its precision (e.g. confidence/credible interval), ideally using structured tables or plots.	10-16
Results of syntheses	20a	For each synthesis, briefly summarise the characteristics and risk of bias among contributing studies.	Not done
	20b	Present results of all statistical syntheses conducted. If meta-analysis was done, present for each the summary estimate and its precision (e.g. confidence/credible interval) and measures of statistical heterogeneity. If comparing groups, describe the direction of the effect.	Not done
	20c	Present results of all investigations of possible causes of heterogeneity among study results.	Not done
	20d	Present results of all sensitivity analyses conducted to assess the robustness of the synthesized results.	Not done
Reporting biases	21	Present assessments of risk of bias due to missing results (arising from reporting biases) for each synthesis assessed.	10-16
Certainty of evidence	22	Present assessments of certainty (or confidence) in the body of evidence for each outcome assessed.	10-16
**DISCUSSION**			
Discussion	23a	Provide a general interpretation of the results in the context of other evidence.	17
	23b	Discuss any limitations of the evidence included in the review.	17
	23c	Discuss any limitations of the review processes used.	17

Section and Topic	Item #	Checklist item	Page where item is reported
	23d	Discuss implications of the results for practice, policy, and future research.	17
**OTHER INFORMATION**			
Registration and protocol	24a	Provide registration information for the review, including register name and registration number, or state that the review was not registered.	3
	24b	Indicate where the review protocol can be accessed, or state that a protocol was not prepared.	3
	24c	Describe and explain any amendments to information provided at registration or in the protocol.	3
Support	25	Describe sources of financial or non-financial support for the review, and the role of the funders or sponsors in the review.	18
Competing interests	26	Declare any competing interests of review authors.	18
Availability of data, code and other materials	27	Report which of the following are publicly available and where they can be found: template data collection forms; data extracted from included studies; data used for all analyses; analytic code; any other materials used in the review.	18

The current checklist followed the latest PRISMA 2020 guideline [1].

Table S2: Keyword and search results in each database

Database	Keyword	Filter	Date*	Result
PubMed	(systemic lupus erythematosus OR SLE) AND (hearing loss OR sensorineural hearing loss OR SNHL OR audiology OR tinnitus OR vertigo OR vestibular OR dizziness OR temporal bone study)	N/A	2024/6/11	258
Embase	(systemic lupus erythematosus OR SLE) AND (hearing loss OR sensorineural hearing loss OR SNHL OR audiology OR tinnitus OR vertigo OR vestibular OR dizziness OR temporal bone study)	N/A	2024/6/11	1919
ClinicalKey	(systemic lupus erythematosus OR SLE) AND (hearing loss OR sensorineural hearing loss OR SNHL OR audiology OR tinnitus OR vertigo OR vestibular OR dizziness OR temporal bone study)	N/A	2024/6/11	563
Web of Science	(systemic lupus erythematosus OR SLE) AND (hearing loss OR sensorineural hearing loss OR SNHL OR audiology OR tinnitus OR vertigo OR vestibular OR dizziness OR temporal bone study)	N/A	2024/6/11	264
ScienceDirect on line	(systemic lupus erythematosus) AND (sensorineural hearing loss OR OR tinnitus OR vertigo OR vestibular)	N/A	2024/6/11	4379

*: initial search date on 2023/12/29, final update on 2024/6/11

Abbreviation: N/A: not applied

Table S3: Excluded studies and reason

Reason	Numbers	References
Review article	5	[2-6]
Mixed autoimmune diseases but not specific systemic lupus erythematosus	1	[7]
Meta-analysis	3	[8-10]
Not related to audiovestibular dysfunction in systemic lupus erythematosus patients	3	[11-13]
Not related to patients with systemic lupus erythematosus	2	[14,15]

Table S4: Newcastle-Ottawa Scale and Characteristics for the Included Trial

	**Selection**				**Comparability**	**Exposure**			**Total**
Study	Case definition	Representative	Control selection	Control definition	Comparability	Ascertainment	Same method	Non-Response rate	Summary
Chen, H. (2022)[16]	*	*	*	*	*	*	*		7*
Polanski, J.F. (2021)[17]	*	*		*	*	*	*		6*
Kim, J.G. (2020)[18]	*					*			2*
Tan, C.L. (2020)[19]	*					*			2*
Bullington, M. (2019)[20]	*					*			2*
Fernandes, M.R.N. (2018)[21]	*					*			2*
Lasso de la Vega, M. (2017)[22]	*	*	*	*	*	*	*		7*
Rahne, T. (2017)[23]	*	*	*	*		*			5*
Chawki, S. (2016)[24]	*					*			2*
Ferrari, A.L. (2016)[25]	*	*				*			3*
Kariya, S. (2016)[26]	*	*	*	*	*	*	*		7*
Kariya, S. (2015)[27]	*	*	*	*	*	*	*		7*

Abbasi, M. (2013)[28]	*	*	*	*	*	*	*			7*
Batuecas-Caletrio, A. (2013)[29]	*	*				*				3*
Garcia-Berrocal, J.R. (2013)[30]	*	*	*	*	*	*	*	*		8*
Lin, C. (2013)[31]	*		*		*	*				4*
Maciaszczyk, K. (2011)[32]	*	*	*	*	*	*	*			7*
Karabulut, H. (2010)[33]	*	*	*	*	*	*	*			7*
Bruner, A.P. (2009)[34]	*	*	*	*	*	*	*			7*
Khalidi, N.A. (2008)[35]	*					*				2*
Gomides, A.P. (2007)[36]	*	*	*	*	*	*	*			7*
Karatas, E. (2007)[37]	*	*	*	*	*	*	*	*		8*
Fukushima, N. (2006)[38]	*	*				*				3*
Roverano, S. (2006)[39]	*	*	*	*	*	*	*			7*
Compadretti, G.C. (2005)[40]	*					*				2*
Cordeschi, S. (2004)[41]	*	*	*	*	*	*	*			7*
Liao, C.H. (2003)[42]	*					*				2*

Jiménez-Alonso, J. (2002)[43]	*	*	*	*	*	*	*	7*
Kastanioudakis, I. (2002)[44]	*	*	*	*		*		5*
Green, L. (2001)[45]	*					*		2*
Sone, M. (1999)[46]	*	*				*		3*
Sperling, N.M. (1998)[47]	*	*				*		3*

* indicated this study have a good performance in this item

Table S5: Summary of the included study

Study	Characteristics			Outcomes	Summary
	Study design	Recruited case characteristics	Number of subjects	Results	Conclusion
Chen, H. (2022)[1]	Case-control	SLE cases with ages ranging from 18 to 65	91 patients and 30 controls	The incidence of hearing loss was 27.47% in SLE patients, significantly higher than in the control group (3.3%) and most cases were mild-to-moderate, bilateral and predominantly sensorineural	HL is not rare in SLE patients, and extended high frequency audiometry can help identify early hearing impairment
Polanski, J.F. (2021)[2]	Case-control	SLE patients with disease duration at least 16 years	43 patients and 41 controls	SLE patients had more sensorineural hearing loss than controls	There is a high prevalence of hearing loss in SLE that is not affected by antimalarial drug use.
Kim, J.G. (2020)[3]	Case report	70-year-old woman with SLE	A female case	Significantly improved vestibulopathy by steroid treatment	As vestibular involvement can herald other systemic presentation of SLE, clinician should be wary in the diagnosis
Tan, C.L. (2020)[4]	Case report	18-year- old male with SLE	A male case	Near-normal hearing was noticed after continuous steroid and hydroxychloroquine. treatment	Early diagnosis and differential diagnosis between others autoimmune disease inner ear diseases are important
Bullington, M. (2019)[5]	Case report	56-year-old woman with SLE	A female case	Steroid treatment completely restore the hearing	Hypertrophic pachymeningitis should be consider in SLE patients with hearing impairment.
Fernandes, M.R.N. (2018)[6]	Case report	51-year-old female with SLE	A female case	Hearing function was not restored after discontinuing hydroxychloroquine	Aim to alert for the ototoxic potential of the antimalarials
Lasso de la Vega, M. (2017)[7]	Case-control	SLE cases with ages ranging from 20 to 60	55 patients and 71 controls	70% cases had sensorineural hearing loss with extended high-frequency audiometry	Sensorineural hearing loss must be considered within the clinical picture of systemic lupus erythematosus

Rahne, T. (2017)[8]	Case-control	SLE cases with ages ranging from 23 to 66	20 patients and 42 controls	A significant correlation between hearing loss and both the cumulative steroid dose and number of organ manifestations	SLE patients are at moderate-to-high risk of conductive hearing loss.
Chawki, S. (2016)[9]	Case report	19-year-old woman with SLE	A female case	Good response to high dosage steroid in aspect of hearing function	Although management remains empirical, the high risk of permanent hearing impairment seems to justify emergency treatment with high-dose corticosteroids
Ferrari, A.L. (2016)[10]	Case series	SLE patients without hearing complaints	89 cases	Asymptomatic sensorineural hearing loss was observed in 14 patients (16%)	Observed asymptomatic sensorineural hearing loss in 16% of SLE and an association with LDL.
Kariya, S. (2016)[11]	Case-control	Histopathology study	8 patients and 10 controls	The area of the stria vascularis in SLE group was significantly smaller than in control group	Provide the histopathologic basis for the cochlear dysfunction, including sensorineural hearing loss, experienced by SLE patients.
Kariya, S. (2015)[12]	Case-control	Histopathology study	8 patients and 17 controls	The mean density of type I hair cells in SLE group was significantly lower than in control group	Our findings could provide a pathologic basis for the difficulty with balance experienced by patients with SLE.
Abbasi, M. (2013)[13]	Case-control	SLE cases with mean age of 34.9 years old	45 patients and 45 controls	Twelve patients (26.7%) in case group and 4 patients (8.9%) in control group had sensorineural hearing loss	SLE patients may develop sensorineural hearing loss during their course of the disease
Batuecas-Caletrio, A. (2013)[14]	Case series	SLE patients with female predominant	89 cases	24% patients reported sensorineural hearing loss or episodic vertigo.	Sensorineural hearing loss and episodic vertigo are comorbid conditions in patients with SLE
Garcia-Berrocal, J.R. (2013)[15]	Case-control	SLE patients with < 1 year disease development	30 patients and 11 controls	No statistically significant audiometric changes between the patients and controls	Asymptomatic hearing loss could be observed over a more extended follow-up period (i.e. more than 10 years)
Lin, C. (2013)[16]	Database research	Database research and no in-person involved patients	7168 patients and 35840 control	Incidence of SSHL was 2.22-fold higher in the SLE group than in the non-SLE group	SLE was significantly associated with an increased risk of developing sensorineural hearing loss

Maciaszczyk, K. (2011)[17]	Case-control	SLE patients with female predominant	35 patients and 30 controls	SLE patients had a significantly poorer mean hearing thresholds than the control group for all frequencies, except for 500; 2000 and 4000 Hz	Auditory system involvement ought to be considered as one of elements of the clinical picture of systemic lupus erythematosus
Karabulut, H. (2010)[18]	Case-control	SLE cases with mean age of 36.3 years old	26 patients and 30 controls	Significant difference in DPOAE and TEOAE between cases and controls	Support a general picture of low frequency hearing loss in systemic lupus erythematosus patients
Bruner, A.P. (2009)[19]	Case-control	SLE patients with or without neuropsychiatry involvement	40 patients and 20 controls	SLE patients presented a significantly lower performance in Temporal Processing tests than controls.	SLE patients presented impaired central auditory processing
Khalidi, N.A. (2008)[20]	Case report	33-year-old woman with SLE	A female case	Poor response to steroid plus azathioprine in aspect of hearing function	Symptomatic sensorineural hearing loss is rare in systemic lupus erythematosus.
Gomides, A.P. (2007)[21]	Case-control	All the subjects were female gender	45 patients and 45 controls	Auditory symptoms were present in 25 (55.5%) patients, with a diagnosis of sensorineural hearing loss in seven (15.6%) patients	Adequate investigation of auditory symptoms is important during the follow-up of patients with SLE
Karatas, E. (2007)[22]	Case-control	SLE cases with female predominant	28 patients and 28 controls	Nineteen (67%) patients reported audiovestibular symptoms. Sensorineural hearing loss was found in 6 (21%) patients	The audiovestibular disturbances in SLE are more prevalent than previously recognized
Fukushima, N. (2006)[23]	Case report	22-year-old woman with SLE	A female case	Observed vasculitis and total damage of the cochlear structures in a patient with SLE.	Direct temporal bone histopathology
Roverano, S. (2006)[24]	Case-control	All the included subjects were female	31 patients and 25 controls	70% cases had impaired hearing, among which 66% cases had sensorineural loss	Significant number of patients with SLE had clinically SNHL at high frequencies, both bilateral and symmetric
Compadretti, G.C. (2005)[25]	Case report	37-year-old woman with SLE	A female case	Successfully treated with osmotic therapy	A hearing deficit may be the first local symptom of an autoimmune disease

Cordeschi, S. (2004)[26]	Case-control	SLE cases with ages ranging from 27 to 66	30 patients and 30 controls	Significantly inverse correlation between duration of disease and TEOAEs amplitude	Confirm a progressive cochlear impairment in SLE patients, though its pathogenesis is still unclear
Liao, C.H. (2003)[27]	Case report	11-year-old boy with SLE	A male case	Good response to high dosage steroid treatment	Physicians should consider the possibility of SLE if patients present with nonspecific neurological symptoms and concomitant systemic symptoms
Jiménez-Alonso, J. (2002)[28]	Case-control	SLE cases with female predominant	91 patients and 87 controls	Sensorineural hearing loss mainly involve middle-high frequency	The frequency of sensorineural hearing loss in SLE patients was higher than in controls
Kastanioudakis, I. (2002)[29]	Case-Control	SLE cases with average age of 47.86 years old	43 patients and 50 controls	22.5% SLE cases had hearing loss; most of them were sensorineural hearing loss.	The mechanism of ear damage remains unknown. Additional prospective studies are needed to elucidate its pathogenesis.
Green, L. (2001)[30]	Case report	22-year-old male with SLE	A male case	Fail to response to steroid treatment in aspect of hearing function	Anticoagulant treatment should be considered, particularly if immunosuppressive therapy is unsuccessful.
Sone, M. (1999)[31]	Case series	SLE cases with disease duration 3 months to 26 years	1 male and 6 female cases	Most of the cases showed a loss of spiral ganglion cells, with various degrees of hair cell loss and atrophy of the stria vascularis	Pathogenesis could be multifactorial, which included influence on pathological conditions and autoimmune response
Sperling, N.M. (1998)[32]	Case series	All the included cases were female	84 cases	Twenty-six (31%) of 84 patients with lupus reported aural symptoms.	Aural symptoms are prevalent among patients with lupus

Abbreviation: DPOAE: distortion product otoacoustic emission; SLE: systemic lupus erythematosus; TEOAE: transient evoked otoacoustic emission

Reference list of supplement tables:

1. Page, M.J.; McKenzie, J.E.; Bossuyt, P.M.; Boutron, I.; Hoffmann, T.C.; Mulrow, C.D.; Shamseer, L.; Tetzlaff, J.M.; Akl, E.A.; Brennan, S.E.; et al. The PRISMA 2020 statement: an updated guideline for reporting systematic reviews. *Bmj* **2021**, *372*, n71, doi:10.1136/bmj.n71.
2. Girasoli, L.; Cazzador, D.; Padoan, R.; Nardello, E.; Felicetti, M.; Zanoletti, E.; Schiavon, F.; Bovo, R. Update on Vertigo in Autoimmune Disorders, from Diagnosis to Treatment. *Journal of immunology research* **2018**, *2018*, 5072582, doi:10.1155/2018/5072582.
3. Riera, J.L.; Del, R.M.M.; Musuruana, J.L.; Cavallasca, J.A. Sudden Sensorineural Hearing Loss in Systemic Lupus Erythematosus and Antiphospholipid Syndrome: A Clinical Review. *Curr Rheumatol Rev* **2020**, *16*, 84-91, doi:10.2174/1573397115666191016101741.
4. Rahne, T.; Plontke, S.; Keysser, G. Vasculitis and the ear: a literature review. *Curr Opin Rheumatol* **2020**, *32*, 47-52, doi:10.1097/BOR.0000000000000665.
5. Stone, J.H.; Francis, H.W. Immune-mediated inner ear disease. *Curr Opin Rheumatol* **2000**, *12*, 32-40, doi:10.1097/00002281-200001000-00006.
6. Di Stadio, A.; Ralli, M. Systemic Lupus Erythematosus and hearing disorders: Literature review and meta-analysis of clinical and temporal bone findings. *J Int Med Res* **2017**, *45*, 1470-1480, doi:10.1177/0300060516688600.
7. Xie, S.; Ning, H.; She, Y.; Jing, Q.; Jiang, Q.; Zhang, Y.; Mei, L.; Feng, Y.; Wu, X. Effect of systemic lupus erythematosus and rheumatoid arthritis on sudden sensorineural hearing loss. *Laryngoscope* **2020**, *130*, 2475-2480, doi:10.1002/lary.28455.
8. Yuen, E.; Fried, J.; Nguyen, S.A.; Rizk, H.G.; Ward, C.; Meyer, T.A. Hearing loss in patients with systemic lupus erythematosus: A systematic review and meta-analysis. *Lupus* **2021**, *30*, 937-945, doi:10.1177/0961203321997919.
9. Paraschou, V.; Chaitidis, N.; Papadopoulou, Z.; Theocharis, P.; Siolos, P.; Festas, C. Association of systemic lupus erythematosus with hearing loss: a systemic review and meta-analysis. *Rheumatol Int* **2021**, *41*, 681-689, doi:10.1007/s00296-021-04788-5.
10. Li, X.; Cao, Z.; Chen, F.; Yang, D.; Zhao, F. Sensorineural Hearing Loss in Autoimmune Diseases: A Systematic Review and Meta-analysis. *J Int Adv Otol* **2023**, *19*, 277-282, doi:10.5152/iao.2023.22991.
11. Poshattiwar, R.S.; Acharya, S.; Shukla, S.; Kumar, S. Neurological Manifestations of Connective Tissue Disorders. *Cureus* **2023**, *15*,

e47108, doi:10.7759/cureus.47108.
12. Motoyama, R.; Higuchi, T.; Hirahara, S.; Konda, N.; Yamada, R.; Watanabe, K.; Fujisaki, M.; Yamaguchi, R.; Katsumata, Y.; Kawaguchi, Y.; et al. A case of systemic lupus erythematosus having concurrent Evans syndrome and acquired thrombotic thrombocytopenic purpura. *Mod Rheumatol Case Rep* **2023**, *7*, 383-387, doi:10.1093/mrcr/rxad011.
13. Crincoli, V.; Piancino, M.G.; Iannone, F.; Errede, M.; Di Comite, M. Temporomandibular Disorders and Oral Features in Systemic Lupus Erythematosus Patients: An Observational Study of Symptoms and Signs. *Int J Med Sci* **2020**, *17*, 153-160, doi:10.7150/ijms.38914.
14. Frejo, L.; Lopez-Escamez, J.A. Cytokines and Inflammation in Meniere Disease. *Clin Exp Otorhinolaryngol* **2022**, *15*, 49-59, doi:10.21053/ceo.2021.00920.
15. Gazquez, I.; Soto-Varela, A.; Aran, I.; Santos, S.; Batuecas, A.; Trinidad, G.; Perez-Garrigues, H.; Gonzalez-Oller, C.; Acosta, L.; Lopez-Escamez, J.A. High prevalence of systemic autoimmune diseases in patients with Meniere's disease. *PloS one* **2011**, *6*, e26759, doi:10.1371/journal.pone.0026759.
16. Chen, H.; Wang, F.; Yang, Y.; Hua, B.; Wang, H.; Chen, J.; Feng, X. Characteristics of Hearing Loss in Patients with Systemic Lupus Erythematosus. *J Clin Med* **2022**, *11*, doi:10.3390/jcm11247527.
17. Polanski, J.F.; Tanaka, E.A.; Barros, H.; Chuchene, A.G.; Miguel, P.T.G.; Skare, T.L. Chloroquine, Hydroxychloroquine and Hearing Loss: A Study in Systemic Lupus Erythematosus Patients. *Laryngoscope* **2021**, *131*, E957-E960, doi:10.1002/lary.28873.
18. Kim, J.G.; Lee, S.U.; Lee, C.N.; Yu, S.W.; Park, K.W.; Kim, J.S. Bilateral vestibulopathy as an early manifestation of systemic lupus erythematosus. *J Neurol* **2020**, *267*, 1855-1858, doi:10.1007/s00415-020-09876-3.
19. Tan, C.L.; Yahaya, M.H.; Ahmad, N.S.; Lim, C.H. Macrophage activation syndrome as an initial presentation of systemic lupus erythematosus with sensorineural hearing loss in a young male patient. *BMJ case reports* **2020**, *13*, doi:10.1136/bcr-2019-233330.
20. Bullington, M.; Davies, G.; MacDonald, C.B. Reversible Sensorineural Hearing Loss Resulting from Hypertrophic Pachymeningitis in Systemic Lupus Erythematosus: A Case Report. *OTO Open* **2019**, *3*, 2473974X19865526, doi:10.1177/2473974X19865526.
21. Fernandes, M.R.N.; Soares, D.B.R.; Thien, C.I.; Carneiro, S. Hydroxychloroquine ototoxicity in a patient with systemic lupus erythematosus. *An Bras Dermatol* **2018**, *93*, 469-470, doi:10.1590/abd1806-4841.20187615.

22. Lasso de la Vega, M.; Villarreal, I.M.; Lopez Moya, J.; Garcia-Berrocal, J.R. Extended high frequency audiometry can diagnose sub-clinic involvement in a seemingly normal hearing systemic lupus erythematosus population. *Acta Otolaryngol* **2017**, *137*, 161-166, doi:10.1080/00016489.2016.1219049.
23. Rahne, T.; Clauss, F.; Plontke, S.K.; Keysser, G. Prevalence of hearing impairment in patients with rheumatoid arthritis, granulomatosis with polyangiitis (GPA, Wegener's granulomatosis), or systemic lupus erythematosus. *Clin Rheumatol* **2017**, *36*, 1501-1510, doi:10.1007/s10067-017-3651-4.
24. Chawki, S.; Aouizerate, J.; Trad, S.; Prinseau, J.; Hanslik, T. Bilateral sudden sensorineural hearing loss as a presenting feature of systemic lupus erythematosus: Case report and brief review of other published cases. *Medicine* **2016**, *95*, e4345, doi:10.1097/MD.0000000000004345.
25. Ferrari, A.L.; Calonga, L.; Lapa, A.T.; Postal, M.; Sinicato, N.A.; Pelicari, K.O.; Peres, F.A.; Valente, J.P.; Soki, M.; Appenzeller, S.; et al. Low-Density Lipoprotein Cholesterol Is Associated With Asymptomatic Sensorineural Hearing Loss in Patients With Systemic Lupus Erythematosus. *J Clin Rheumatol* **2016**, *22*, 312-315, doi:10.1097/RHU.0000000000000382.
26. Kariya, S.; Kaya, S.; Hizli, O.; Hizli, P.; Nishizaki, K.; Paparella, M.M.; Cureoglu, S. Cochlear Histopathologic Findings in Patients With Systemic Lupus Erythematosus: A Human Temporal Bone Study. *Otol Neurotol* **2016**, *37*, 593-597, doi:10.1097/MAO.0000000000001017.
27. Kariya, S.; Hizli, O.; Kaya, S.; Hizli, P.; Nishizaki, K.; Paparella, M.M.; Cureoglu, S. Histopathologic Findings in Peripheral Vestibular System From Patients With Systemic Lupus Erythematosus: A Human Temporal Bone Study. *Otol Neurotol* **2015**, *36*, 1702-1707, doi:10.1097/MAO.0000000000000897.
28. Abbasi, M.; Yazdi, Z.; Kazemifar, A.M.; Bakhsh, Z.Z. Hearing loss in patients with systemic lupus erythematosus. *Glob J Health Sci* **2013**, *5*, 102-106, doi:10.5539/gjhs.v5n5p102.
29. Batuecas-Caletrio, A.; del Pino-Montes, J.; Cordero-Civantos, C.; Calle-Cabanillas, M.I.; Lopez-Escamez, J.A. Hearing and vestibular disorders in patients with systemic lupus erythematosus. *Lupus* **2013**, *22*, 437-442, doi:10.1177/0961203313477223.
30. Garcia-Berrocal, J.R.; De Diego, B.; Roldan-Fidalgo, A.; Yebra-Bango, M.; Millan, I.; Trinidad, A.; Ramirez-Camacho, R. Young systemic

lupus erythematosus patients with no hearing involvement: 10-year follow up. *J Laryngol Otol* **2013**, *127*, 38-42, doi:10.1017/S0022215112002769.
31. Lin, C.; Lin, S.W.; Weng, S.F.; Lin, Y.S. Risk of sudden sensorineural hearing loss in patients with systemic lupus erythematosus: a population-based cohort study. *Audiol Neurootol* **2013**, *18*, 95-100, doi:10.1159/000345512.
32. Maciaszczyk, K.; Durko, T.; Waszczykowska, E.; Erkiert-Polguj, A.; Pajor, A. Auditory function in patients with systemic lupus erythematosus. *Auris Nasus Larynx* **2011**, *38*, 26-32, doi:10.1016/j.anl.2010.04.008.
33. Karabulut, H.; Dagli, M.; Ates, A.; Karaaslan, Y. Results for audiology and distortion product and transient evoked otoacoustic emissions in patients with systemic lupus erythematosus. *J Laryngol Otol* **2010**, *124*, 137-140, doi:10.1017/S0022215109991332.
34. Bruner, A.P.; Sato, E.I.; Pereira, L.D. Central auditory processing in patients with systemic lupus erythematosus. *Acta reumatologica portuguesa* **2009**, *34*, 600-607.
35. Khalidi, N.A.; Rebello, R.; Robertson, D.D. Sensorineural hearing loss in systemic lupus erythematosus: case report and literature review. *J Laryngol Otol* **2008**, *122*, 1371-1376, doi:10.1017/S0022215108001783.
36. Gomides, A.P.; do Rosario, E.J.; Borges, H.M.; Gomides, H.H.; de Padua, P.M.; Sampaio-Barros, P.D. Sensorineural dysacusis in patients with systemic lupus erythematosus. *Lupus* **2007**, *16*, 987-990, doi:10.1177/0961203307084160.
37. Karatas, E.; Onat, A.M.; Durucu, C.; Baglam, T.; Kanlikama, M.; Altunoren, O.; Buyukhatipoglu, H. Audiovestibular disturbance in patients with systemic lupus erythematosus. *Otolaryngol Head Neck Surg* **2007**, *136*, 82-86, doi:10.1016/j.otohns.2006.06.1255.
38. Fukushima, N.; Fukushima, H.; Cureoglu, S.; Schachern, P.A.; Paparella, M.M. Hearing loss associated with systemic lupus erythematosus: temporal bone histopathology. *Otol Neurotol* **2006**, *27*, 127-128, doi:10.1097/01.mao.0000201822.49187.a6.
39. Roverano, S.; Cassano, G.; Paira, S.; Chiavarini, J.; Graf, C.; Rico, L.; Heredia, C. Asymptomatic sensorineural hearing loss in patients with systemic lupus erythematosus. *J Clin Rheumatol* **2006**, *12*, 217-220, doi:10.1097/01.rhu.0000242777.71604.69.
40. Compadretti, G.C.; Brandolini, C.; Tasca, I. Sudden sensorineural hearing loss in lupus erythematosus associated with antiphospholipid syndrome: case report and review. *Ann Otol Rhinol Laryngol* **2005**, *114*, 214-218, doi:10.1177/000348940511400308.
41. Cordeschi, S.; Salvinelli, F.; D'Ascanio, L. Sensorineural hearing impairment in systemic lupus erythematosus: sudden or progressive?

Clinical and experimental rheumatology **2004**, *22*, 653.
42. Liao, C.H.; Yang, Y.H.; Chiang, B.L. Systemic lupus erythematosus with presentation as vertigo and vertical nystagmus: report of one case. *Acta Paediatr Taiwan* **2003**, *44*, 158-160.
43. Jimenez-Alonso, J.; Gutierrez-Cabello, F.; Castillo, J.L.; Sabio, J.M.; Hidalgo-Tenorio, C.; Leon, L.; Grupo Lupus Virgen de las, N. Ear involvement in systemic lupus erythematosus patients: a comparative study. J Laryngol Otol 116:103-7. *J Laryngol Otol* **2002**, *116*, 746, doi:10.1258/002221502760238127.
44. Kastanioudakis, I.; Ziavra, N.; Voulgari, P.V.; Exarchakos, G.; Skevas, A.; Drosos, A.A. Ear involvement in systemic lupus erythematosus patients: a comparative study. *J Laryngol Otol* **2002**, *116*, 103-107, doi:10.1258/0022215021910032.
45. Green, L.; Miller, E.B. Sudden sensorineural hearing loss as a first manifestation of systemic lupus erythematosus: association with anticardiolipin antibodies. *Clin Rheumatol* **2001**, *20*, 220-222, doi:10.1007/s100670170069.
46. Sone, M.; Schachern, P.A.; Paparella, M.M.; Morizono, N. Study of systemic lupus erythematosus in temporal bones. *Ann Otol Rhinol Laryngol* **1999**, *108*, 338-344, doi:10.1177/000348949910800404.
47. Sperling, N.M.; Tehrani, K.; Liebling, A.; Ginzler, E. Aural symptoms and hearing loss in patients with lupus. *Otolaryngol Head Neck Surg* **1998**, *118*, 762-765, doi:10.1016/S0194-5998(98)70265-7.
